# Risk Factor Assessment and Predictive Modeling for Ventilator‐Associated Pneumonia: Design and Clinical Implementation of an Artificial Intelligence‐Enhanced Early Detection Framework Using Multisource Data Analytics

**DOI:** 10.1111/crj.70144

**Published:** 2025-11-29

**Authors:** Jia Zhang, Yitong Wang, Yuwei Cao, Jiaxin Li, Shuangmei Dai, Yulin Li, Zhe Zhang, Xin Zhang, Rui Yang, Xinjun Zhang, Jichao Chen, Wailong Zou

**Affiliations:** ^1^ Department of Respiratory and Critical Care Medicine Aerospace Center Hospital Beijing China; ^2^ First Department of Gerontology Aerospace Center Hospital Beijing China

**Keywords:** clinical practice, computed tomography, decision support systems, machine learning, retrospective study, risk factors, ventilator‐associated pneumonia

## Abstract

**Introduction:**

Ventilator‐associated pneumonia (VAP) is associated with poor patient outcomes. Early identification of high‐risk patients remains a major clinical challenge. We aimed to develop and validate a multimodal hybrid neural network (MM‐HNN) for improved VAP prediction by integrating multisource data from a retrospective cohort.

**Methods:**

This single‐center, retrospective study analyzed data from 213 adult patients who received invasive mechanical ventilation for >48 h. The MM‐HNN incorporated three data types: 1) computed tomography (CT) features quantifying consolidation volume through three‐dimensional residual neural network‐50; 2) dynamic ventilator parameters including fraction of inspired oxygen and positive end‐expiratory pressure analyzed via long short‐term memory networks; and 3) clinical predictors refined via least absolute shrinkage and selection operator regression to identify six key variables.

**Results:**

The model achieved an area under the curve of 0.86 (95% confidence interval: 0.80–0.91), outperforming the clinical pulmonary infection score (*p* = 0.021). SHapley Additive exPlanation analysis revealed Acute Physiology and Chronic Health Evaluation II score and CT consolidation volume as primary contributors. The system provided early warnings with 87.5% accuracy (median lead time: 1.5 days), which was associated with a significant increase in appropriate antibiotic use from 68.3% to 92.1% (*p* = 0.016).

**Conclusion:**

The MM‐HNN demonstrates the feasibility of accurate, interpretable VAP risk prediction through multimodal data integration. This artificial intelligence framework provides a clinically actionable tool for dynamic risk assessment, enabling preemptive interventions and improved antibiotic stewardship.

## Graphical abstract

This study developed an interpretable multimodal hybrid neural network integrating computed tomography radiomics, ventilator dynamics, and clinical indicators to predict ventilator‐associated pneumonia. The model achieved an area under curve of 0.86, enabled early warnings at 1.5 days prediagnosis, and increased appropriate antibiotic use rates by 23.8% (68.3% → 92.1%). This model demonstrates the AI‐enhanced optimization of antimicrobial stewardship in critical care.

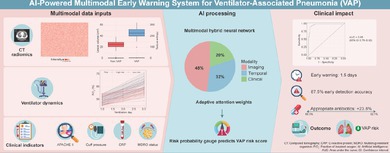

## 1 Introduction

Ventilator‐associated pneumonia (VAP), a severe complication in patients on mechanical ventilation, contributes to increased morbidity, mortality, and healthcare costs. It markedly affects patients and poses a heavy burden on intensive care units [1]. Early and accurate identification of patients at high risk for VAP remains a key clinical challenge. Conventional diagnostic methods relying on subjective indicators lack dynamic risk assessment capabilities, failing to meet early warning demands [2]. The rapid advancement of artificial intelligence (AI) technologies offers novel solutions for integrating multisource heterogeneous medical data, such as imaging features, temporal physiological parameters, and clinical indicators, to construct intelligent diagnostic models. These models have substantial potential to overcome VAP early detection barriers, optimize clinical decision‐making, and reduce healthcare costs [3].

Globally, although AI applications in pulmonary disease diagnosis have been explored [4], existing VAP studies primarily focus on single‐data modalities or conventional statistical models. This approach often overlooks systematic integration of key factors, such as dynamic mechanical ventilation management parameters (including cuff pressure monitoring), host–pathogen interaction characteristics, and quantitative imaging biomarkers [5]. Although domestic research has achieved algorithmic progress [6], critical gaps remain in multimodal data fusion modeling, model interpretability, and clinical validation. Specifically, insufficient exploration of VAP's complex pathogenic mechanisms—such as the dynamic correlation between ventilation duration and infection risk—limits model generalizability and clinical utility [7].

The three main components employed in our multimodal hybrid neural network (MM‐HNN)—namely, three‐dimensional computed tomography (CT) imaging, dynamic ventilator parameters (cuff pressure, positive end‐expiratory pressure [PEEP], and fraction of inspired oxygen [FiO_2_]), and key clinical indicators (Acute Physiology and Chronic Health Evaluation II [APACHE II] score, cuff pressure failure frequency, and CT consolidation ≥5 cm^3^)—constitute a solid foundation of predictors. This study design was informed by a comprehensive review of VAP pathogenesis and prior evidence. We initially collected a set of 32 candidate variables, including the core features presented in the final model and factors such as microbiological colonization history, inflammatory biomarkers (e.g., C‐reactive protein [CRP] and procalcitonin level peaks and trends), detailed ventilation duration and associated events, and preventive care practices (e.g., oral care frequency, head‐of‐bed elevation). The selection of the final predictor set was driven by results of least absolute shrinkage and selection operator (LASSO) regression to identify the most parsimonious and impactful set of variables, balancing predictive power with clinical practicality and mitigating the risk of overfitting in our cohort, rather than being driven by an a priori exclusion of these clinically relevant factors.

We aimed to explore the development and feasibility of an early VAP warning system through multidimensional data fusion and AI technologies using a retrospective single‐center cohort. The exploratory innovations include several key components as follows:

### 1.1 Multimodal Fusion Architecture

A prototype approach investigating the integration of CT‐based quantitative imaging analysis, real‐time ventilator parameter monitoring, and structured clinical indicators via adaptive weight allocation, which aims to address the limitations of single‐modality models for enhanced diagnostic accuracy in this context [8]. This integration must account for potential inaccuracies inherent in retrospective electronic health record (EHR) data.

### 1.2 Dynamic Risk Stratification

An investigation combining risk factor screening with time‐varying risk analysis within the available data to identify critical dynamic metrics, such as cuff pressure maintenance and PEEP stability, as features for predictive modeling [9]. It is recognized that clinician management decisions are crucial confounding factors influencing observed outcomes.

### 1.3 Enhanced Model Interpretability

By employing methods such as visualizing feature contributions (e.g., SHapley Additive exPlanations [SHAP]) and constructing case similarity networks, this approach seeks to elucidate associations between imaging biomarkers and microbial colonization, aiming to provide interpretable insights for clinicians less familiar with complex AI models [10].

### 1.4 Proof‐of‐Concept Implementation

Establishing an initial comprehensive intelligent system that integrates risk factor discovery and multimodal modeling demonstrates potential utility [11].

## 2 Materials and Methods

### 2.1 Data Sources and Collection

This retrospective single‐center case–control study utilized de‐identified EHRs from a tertiary hospital in Beijing, China, with no direct patient participation or intervention. The study met criteria for exemption of ethical review under U.S. Federal Regulation 45 CFR §46.104(d) (4)(ii) [12]. Ethical approval was granted with a waiver of informed consent by the Ethics Committee of Aerospace Center Hospital (Approval No. JHYK‐EC2024‐090). Data collection adhered to China's Information Security Technology—Health Data Security Guidelines (GB/T 39725‐2020) [13] and the Helsinki Declaration. Due to the exclusive reliance on EHRs, potential inaccuracies in data entry (e.g., manual documentation errors) and interpretation biases were monitored through rigorous quality control.

### 2.2 Data Sources and Study Limitations

A multimodal dataset was retrospectively extracted from five clinical information systems at Aerospace Center Hospital (Table 1).

**TABLE 1:** Multimodal dataset construction from clinical information systems.

System	Extracted variables	Data format
Hospital information system (HIS)	Demographics (age, sex, BMI), comorbidities (COPD, diabetes, immunosuppression), APACHE II score, treatment regimens	Structured data
ICU monitoring system (ICIS)	Ventilator parameters (mode, PEEP levels, total ventilation duration), blood gas analysis (PaO₂/FiO₂), vital sign trends	Time‐series data
Laboratory system (LIMS)	Microbiological culture results (sputum/BALF), antimicrobial susceptibility, inflammatory markers (PCT, CRP, WBC)	Semistructured reports
Imaging archiving system (PACS)	Chest X‐ray/CT features (new infiltrates, consolidation extent), radiology reports	DICOM files + narrative text
Pharmacy system (EDMS)	Antibiotic usage (type, initiation time, duration), PPIs, cumulative sedative/muscle relaxant doses	Time–event sequence data

Abbreviations: APACHE II, acute physiology and chronic health evaluation II; BALF, bronchoalveolar lavage fluid; CRP, C‐reactive protein; COPD, chronic obstructive pulmonary disease; DICOM, Digital Imaging and Communications in Medicine; FiO_2_, fraction of inspired oxygen; ICIS, Integrated Clinical Information System; LIMS, Laboratory Information Management System; PaO_2_, partial pressure of oxygen; PACS, Picture Archiving and Communication System; PCT, procalcitonin; PEEP, positive end‐expiratory pressure; PPI, proton pump inhibitor; WBC, white blood cell count.

### 2.3 Cohort Selection Process

#### 2.3.1 Inclusion Criteria

**FIGURE 1:**
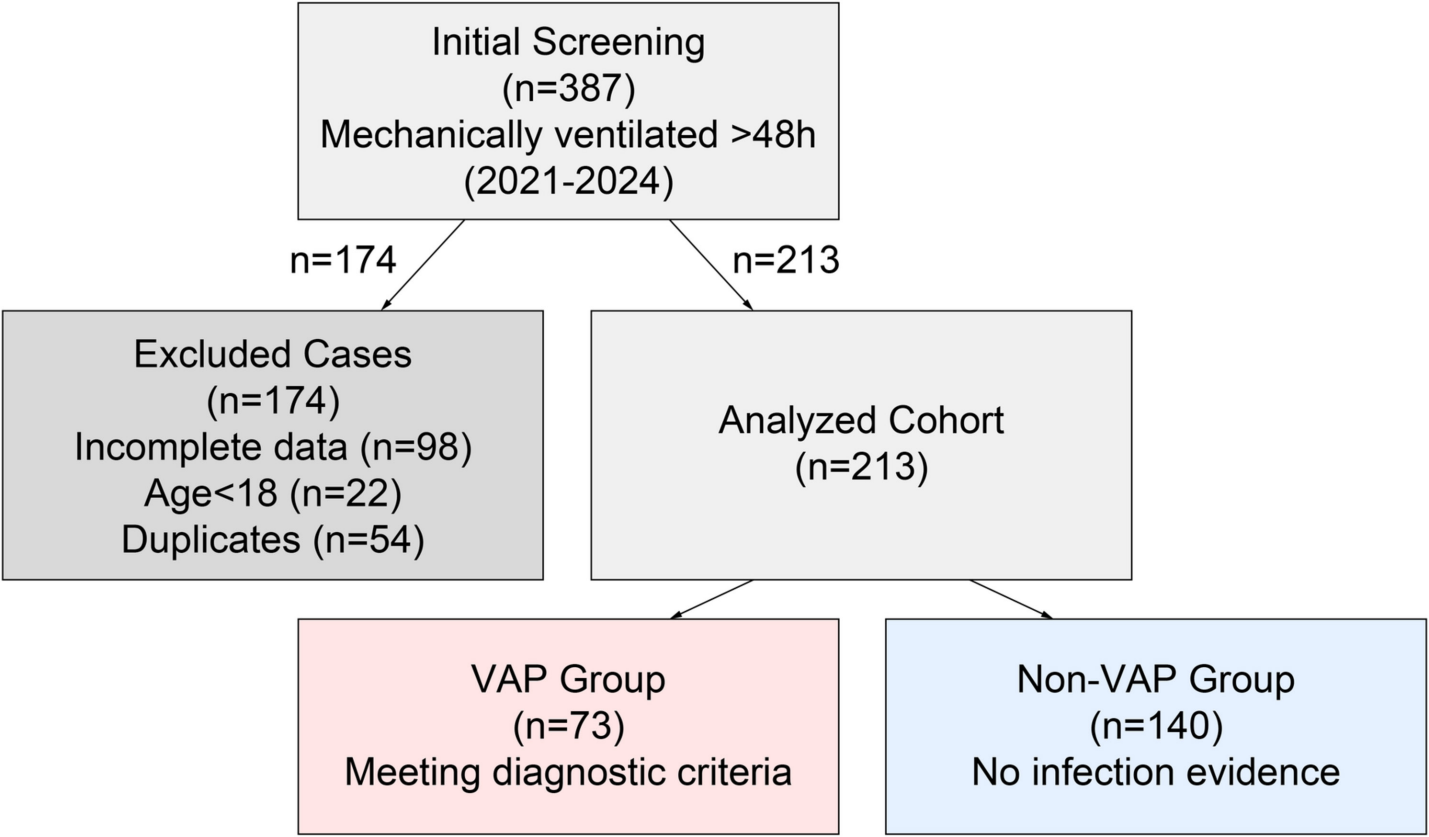
Study population screening and grouping workflow. (1) VAP diagnosis requires concurrent fulfilment of both imaging and clinical criteria. (2) “Duplicate entries” denote repeated records from the same patient due to interdepartmental transfers. (3) Gray arrows indicate data cleaning workflows, whereas red and blue frames differentiate case groupings.

- Patients admitted to intensive care unit (ICU)/Aerospace Center Hospital (January 2021–March 2024).
- Age ≥18 years (Figure 1).

Invasive mechanical ventilation >48 h.

#### 2.3.2 Exclusion Criteria

- Critical data gaps (>20% absence in ventilation/microbiology records).
- Preexisting infection or death <48 h postadmission.

Palliative care/do not resuscitate orders.

#### 2.3.3 Final Cohort

- Initial screening: 387 patients.
- Exclusions: 174 (incomplete data: 98; age: 22; duplicates: 54).
- Analyzed cohort: 213 patients (single center).

VAP group: *n* = 73 (diagnosed per Infectious Diseases Society of America/American Thoracic Society [IDSA/ATS] criteria [14]).

○ Non‐VAP group: *n* = 140 (ventilation duration matched, ±10%).

### 2.4 Data Collection Protocol

#### 2.4.1 Candidate Risk Factors

Our study was designed to capture a comprehensive set of potential predictors based on VAP pathogenesis guidelines [14], prior evidence, and multidisciplinary consensus from experts from the Department of Pulmonary and Critical Care Medicine and Department of Radiology and from specialists in infectious diseases (ID) and medical informatics. We initially collected and evaluated 32 candidate variables across three broad domains, which included not only the core features presented in the final model but also factors, such as microbiological colonization history, inflammatory biomarkers (e.g., CRP and procalcitonin [PCT] level peaks and trends), detailed ventilation duration and associated events, and preventive care practices (e.g., oral care frequency, head‐of‐bed elevation). These were finalized through multidisciplinary consensus across three domains:

- Baseline: age, comorbidities (diabetes, immunosuppression).
- Treatment: ventilation parameters, antibiotics.
- Biomarkers: inflammatory markers/radiomics.

The subsequent refinement of this broad dataset into a parsimonious set of core predictors was achieved through rigorous statistical feature selection, as detailed in the “Statistical Analysis Framework Design” section below.

#### 2.4.2 Data Collection Team

Attending physicians, nurses, informatics staff.

#### 2.4.3 Quality Control

- Dual daily recordings with mean/frequency validation.
- Systemic checks for EHR inconsistencies (e.g., outlier values in cuff pressure logs).

### 2.5 Core Data Elements

#### 2.5.1 Demographics and Severity

- Age, sex, body mass index (BMI), APACHE II score (24‐h preventilation peak).
- Comorbidities (diabetes, chronic obstructive pulmonary disease), admission diagnosis.

#### 2.5.2 Treatment Variables

- Ventilation: intubation route, mean cuff pressure, frequency of cuff pressure <20 cmH_2_O, PEEP/FiO_2_ dynamics.
- Infection control: oral care frequency, nurse‐documented head elevation compliance.
- Pharmacotherapy: antibiotic class/duration, sedative dosing.

#### 2.5.3 Clinical/Laboratory Metrics

- Symptoms: temperature peaks, crackles/wheezes (nursing notes), sputum purulence.
- Labs: CRP/PCT level peaks, partial pressure of oxygen/FiO₂ nadir, microbiology timing/resistance profiles (e.g., carbapenem‐resistant *Acinetobacter baumannii* ).
- Imaging: CT/X‐ray infiltrates (radiologist‐blinded review), consolidation volume.

### 2.6 Outcome Definitions

#### 2.6.1 Primary Outcome (VAP)

1. New/progressive infiltrates >48 h postintubation.
2. Clinical signs ≥2 (fever, purulent sputum, white blood cell abnormality).
3. Microbiological confirmation per IDSA/ATS [14].

- Secondary: ventilation/ICU days, 28‐day mortality.
- Confounders adjusted: APACHE II score, comorbidities, preventilation antibiotics.
- VAP onset time definition: to precisely anchor the prediction timeline, VAP onset was rigorously defined as the earliest timestamp at which all three diagnostic components were definitively documented in the EHR. This typically corresponded to either the microbiological culture order or the imaging report confirming infiltrates, whichever occurred later, following the onset of clinical signs. Consistent with methodologies that use clinical actions to mark event onset, this approach ensures that the model's predictions target the period prior to when the combination of findings prompts clinical suspicion and diagnostic confirmation.
- Lead time calculation: the reported median alert time advancement was calculated as the difference between the time of the model's first risk alert exceeding the predefined threshold and the objectively defined VAP onset time.

### 2.7 Data Quality Control and Limitations

#### 2.7.1 Quality Assurance

- Automated EHR checks excluded 22 cases.
- Random verification (30%; *n* = 64) showed <5% variance.
- Expert adjudication for 15 disputed cases.

Methodological note: uncontrolled confounding may exist due to undocumented variations in clinical decision‐making (e.g., sedative titration protocols, empiric antibiotic selection), an inherent limitation of retrospective observational designs.

#### 2.7.2 Missing Data

- Noncritical (<10% missing): multiple imputation.
- Critical missing (e.g., culture/blood gas): exclusion.

#### 2.7.3 Standardization

- Timestamp synchronization (<1 min deviation).
- SI unit conversion for laboratory values.

### 2.8 Security and Generalizability Note

- Anonymized/encrypted storage with audit trails.

## 3 Statistical Analysis Framework Design

### 3.1 Risk Factor Screening and Analysis

#### 3.1.1 Core Variable Identification

- Univariate screening: analyzed 32 candidate predictors using independent *t*‐tests (continuous variables) or chi‐square/Fisher's exact tests (categorical variables). Significance threshold: *p* < 0.05, post‐Benjamini–Hochberg correction.
- Multivariate modeling: LASSO regression (*λ* = 0.1) was employed to refine the initial broad set of predictors and identify key variables, followed by logistic regression to estimate standardized coefficients (*β*) and odds ratios (ORs, 95% confidence interval [CI]).

### 3.2 Dynamic Risk Stratification

Time‐varying risk assessment: Cox models assessed risk evolution at ventilation days 3, 5, and 7.

### 3.3 Multimodal Prediction Modeling

#### 3.3.1 Data Fusion Strategy

- Imaging: three‐dimensional (3D) residual neural network (ResNet‐50) extracted CT consolidation volumes and texture features.
- Temporal data: long short‐term memory (LSTM) networks captured ventilator parameter trends.
- Structured data: eXtreme Gradient Boosting (XGBoost) optimized clinical feature subsets (e.g., CRP level, PCT, antibiotic days).

#### 3.3.2 Model Architecture

MM‐HNN with multihead attention dynamically weighted features:

$$\text{Attention}\;\left(Q,K,V\right)=\text{Softmax}\left[\left({\mathrm{QK}}^{T}\right)/\surd d_{k}\right]V,$$

where query (Q), key (K), value (V) vectors are derived from imaging, temporal, and clinical embeddings, with *d*_k_ as a scaling factor.

### 3.4 Model Validation and Interpretation

#### 3.4.1 Performance Metrics

- Discrimination: receiver operating characteristic–area under the curve (ROC‐AUC).
- Calibration: Brier score [15].
- Clinical utility: decision curve analysis (DCA) for net benefit.

#### 3.4.2 Explainability

- Global: SHAP values ranked feature contributions.
- Local: case similarity networks visualized patient‐specific decision rationales via Python.

### 3.5 Implementation and Reporting

- Software: R 4.3.1 (traditional stats); Python 3.10 (scikit‐learn 1.2.2 for preprocessing); PyTorch 2.0.1 (neural networks).
- Reporting: TRIPOD‐compliant; continuous variables presented as mean ± standard deviation or median (interquartile range [IQR]); categorical variables as counts (%).
- Data security: hosted on hospital‐secured servers (GB/T 39725‐2020 [13]); code access requires confidentiality disclosure agreement/ethics approval.

## 4 Results

### 4.1 Risk Factor Screening and Analysis

#### 4.1.1 Core Variable Identification

Univariate analysis of 213 patients (VAP group, 73; non‐VAP group, 140) was conducted using the following:

- Continuous variables: (age, APACHE II score, frequency of inadequate cuff pressure) were analyzed using the Mann–Whitney *U* test (nonnormal distribution).
- Categorical variables: (sex, comorbidities, multidrug‐resistant organism [MDRO] colonization) were assessed with the chi‐square or Fisher's exact test; cell counts were <5.
- Multiple testing correction: Benjamini–Hochberg method (false discovery rate [FDR] < 0.05).
  Among 32 candidate predictors, 9 retained significance post‐FDR correction (Table 2).

**TABLE 2:** Significant risk factors post‐FDR adjustment.

Variable	VAP group (*n* = 73)	Non‐VAP group (*n* = 140)	Statistic	Raw *p*	Adjusted *p* (FDR)
APACHE II score	18.3 ± 4.1	15.2 ± 3.8	*U* = 3256.5	<0.001	<0.001*
Cuff pressure <20 cmH_2_O frequency	3.2 ± 1.5/day	1.8 ± 1.1/day	*U* = 2890.2	0.002	0.016*
CT consolidation ≥5 cm^3^	56.2% (41/73)	22.9% (32/140)	*χ* ^2^ = 23.1	<0.001	<0.001*
MDRO colonization	41.1% (30/73)	12.1% (17/140)	Fisher	<0.001	0.004*
Acid suppression use	68.5% (50/73)	42.1% (59/140)	*χ* ^2^ = 12.8	<0.001	0.008*
Delayed enteral nutrition (>48 h)	58.9% (43/73)	35.0% (49/140)	*χ* ^2^ = 10.3	0.001	0.024*
Peak CRP (mg/L)	152.3 ± 56.7	98.5 ± 42.1	*U* = 2987.4	<0.001	0.012*
PEEP variability (cmH_2_O)	4.2 ± 1.8	2.6 ± 1.2	*U* = 3012.7	0.003	0.018*
Purulent sputum	78.1% (57/73)	45.7% (64/140)	*χ* ^2^ = 18.6	<0.001	0.006*

Continuous variables: mean ± SD; categorical variables: % (*n*).

Statistics: *U* (Mann–Whitney *U*), *χ* ^2^ (chi‐square), Fisher (exact test).

*FDR‐adjusted significance (FDR < 0.05).

#### 4.1.2 Key Findings

- Dynamic treatment factors: inadequate cuff pressure frequency (*p* = 0.016) and PEEP variability (*p* = 0.018) demonstrated strong VAP associations, underscoring the critical role of consistent ventilator management in infection prevention.
- Host–pathogen factors: the APACHE II score, MDRO colonization, and peak CRP level correlated with disease severity (*p* < 0.01), highlighting that both baseline patient vulnerability and active infectious processes are key drivers of VAP risk.
- Imaging biomarker: CT consolidation ≥5 cm^3^ showed the highest predictive value (OR = 2.18, 95% CI: 1.58–2.67), suggesting that quantitative imaging may be a more objective and powerful indicator than traditional radiographic assessments alone.

### 4.2 Multivariable Modeling

#### 4.2.1 LASSO‐Based Variable Selection

Among nine univariate‐significant predictors, LASSO regression (*λ* = 0.1) identified six independent predictors: APACHE II score, frequency of inadequate cuff pressure, CT consolidation volume ≥5 cm^3^, acid suppressant use, peak CRP level (mg/L), and MDRO positivity (Table 3).

**TABLE 3:** Multivariable logistic regression results.

Variable	Standardized *β*	OR (95% CI)	*p*
APACHE II score (per 1‐point increase)	0.83	1.12 (1.08–1.16)	<0.001
Cuff pressure <20 cmH_2_O (per episode/day)	0.61	1.83 (1.42–2.36)	0.004
CT consolidation ≥5 cm^3^ (yes vs. no)	0.78	2.18 (1.58–2.67)	0.008
Acid suppressant use (yes vs. no)	0.52	1.68 (1.25–2.26)	0.015
Peak CRP (per 10 mg/L increase)	0.47	1.07 (1.02–1.12)	0.021
MDRO positivity (yes vs. no)	0.65	2.34 (1.65–3.32)	0.002

Abbreviation: MDRO, multidrug‐resistant organisms.

#### 4.2.2 Key Findings

- Disease severity: each 1‐point increase in the APACHE II score conferred 12% higher VAP risk (OR = 1.12, *p* < 0.001), emphasizing the need for heightened vigilance in more critically ill patients throughout their ventilation course.
- Ventilation management: daily episodes of inadequate cuff pressure increased VAP risk by 83% (OR = 1.83, *p* = 0.004), providing quantitative evidence for the importance of rigorous, continuous cuff pressure–monitoring protocols.
- Imaging/infection: CT consolidation ≥5 cm^3^ (OR = 2.18) and MDRO positivity (OR = 2.34) were strong independent predictors. This synergy between radiological evidence and microbiological status offers a composite view of infection severity and resistance profile.
- Treatment exposure: acid suppressants increased risk by 68% (OR = 1.68, *p* = 0.015), which may reflect the unintended consequence of altering gastric pH and promoting pathogen colonization, suggesting a need for judicious use.
- Inflammation: each 10 mg/L CRP level elevation increased the risk by 7% (OR = 1.07, *p* = 0.021), positioning serial CRP level measurement as a valuable tool for tracking infection progression and response to therapy.
- Pathogen profile: compared with noncarriers, MDRO carriers had a 134% increased risk (OR = 2.34, *p* = 0.002), flagging these patients as requiring more aggressive diagnostic and preventive strategies from the outset.

#### 4.2.3 VAP Prediction Equation

$\begin{array}{c} \text{logit}\left(p\right)=-4.2+0.83\cdot X₁+0.61\cdot X₂+0.78\cdot X₃+0.52\cdot X₄+0.47\cdot X₅+ \end{array}\begin{array}{c} 0.65\cdot X₆ \end{array}$

#### 4.2.4 Variable Definitions

- *X*_1_: APACHE II score (per 1‐point increase).
- *X*_2_: daily cuff pressure inadequacy episodes.
- *X*_3_: CT consolidation ≥5 cm^3^ (1 = yes, 0 = no).
- *X*_4_: acid suppressant use (1 = yes, 0 = no).
- *X*_5_: peak CRP level (per 10 mg/L increase).
- *X*_6_: MDRO positivity (1 = yes, 0 = no).

#### 4.2.5 Interpretation

- Intercept (*β*_0_ = −4.2): baseline log‐odds of VAP risk.
- Standardized coefficients (*β*_1_–*β*_6_): quantify independent contributions to risk stratification.

#### 4.2.6 Probability Calculation

The probability of VAP occurrence denoted as *p* is computed as: *p* = 1/[1 + *e* ^−logit(*p*)^]. This converts the linear combination of predictors (logit) into a constrained value within the interval 0 < *p* < 1.

### 4.3 Dynamic Risk Assessment

Analysis using the time‐varying Cox model demonstrated that each additional day of mechanical ventilation significantly increased the hazard ratio (HR) for VAP, with temporal trends as follows:

- Day 3: HR = 1.8 (95% CI: 1.2–2.7).
- Day 5: HR = 3.1 (95% CI: 2.0–4.9).
- Day 7: HR = 4.5 (95% CI: 3.0–6.8).

**FIGURE 2:**
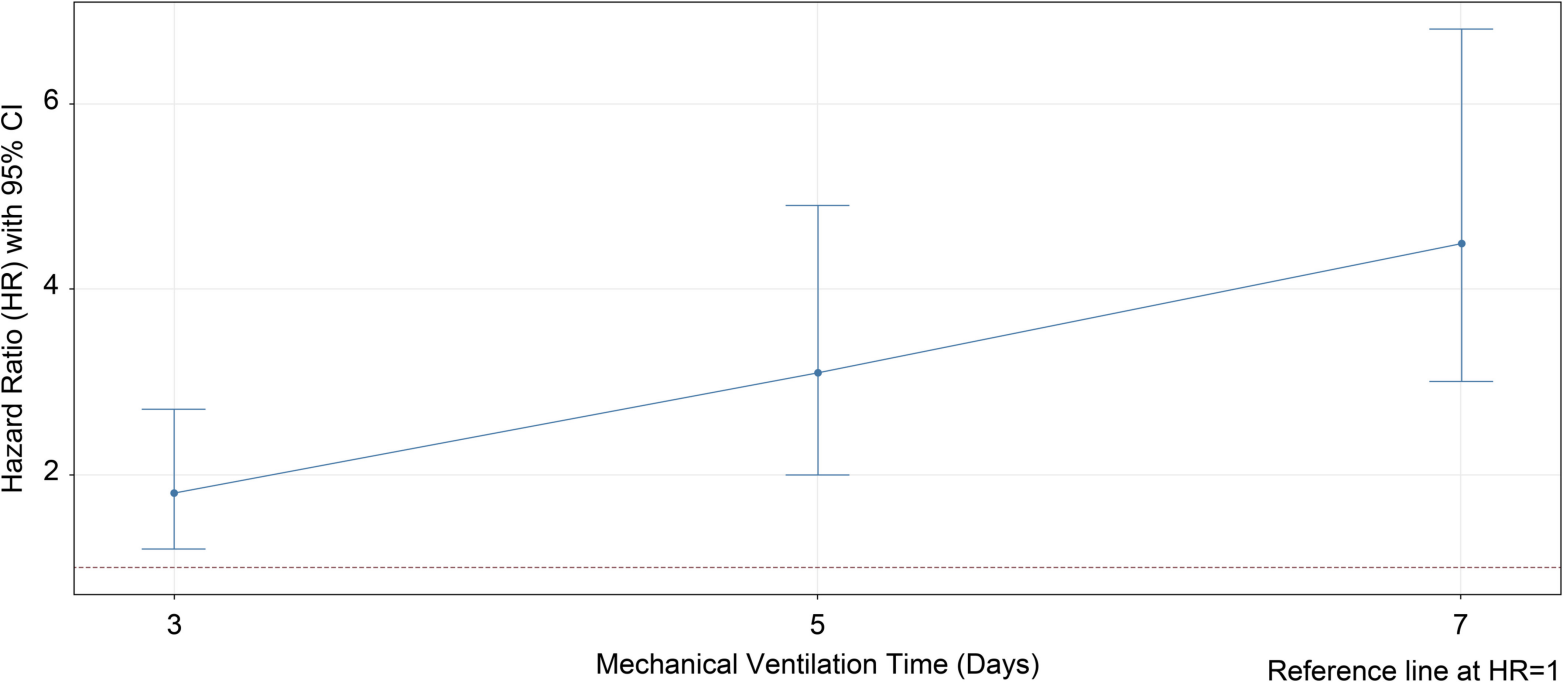
Forest plot of time‐varying Cox model analysis. Forest plot of time‐varying Cox model analysis showing the association between ventilation duration and VAP risk.

These results demonstrate a dose‐dependent escalation of VAP risk with prolonged ventilation duration (Figure 2), quantifying the imperative for early extubation and validating the clinical focus on reducing ventilator days.

### 4.4 Multimodal Prediction Model Development

#### 4.4.1 Data Fusion Protocol

**FIGURE 3:**
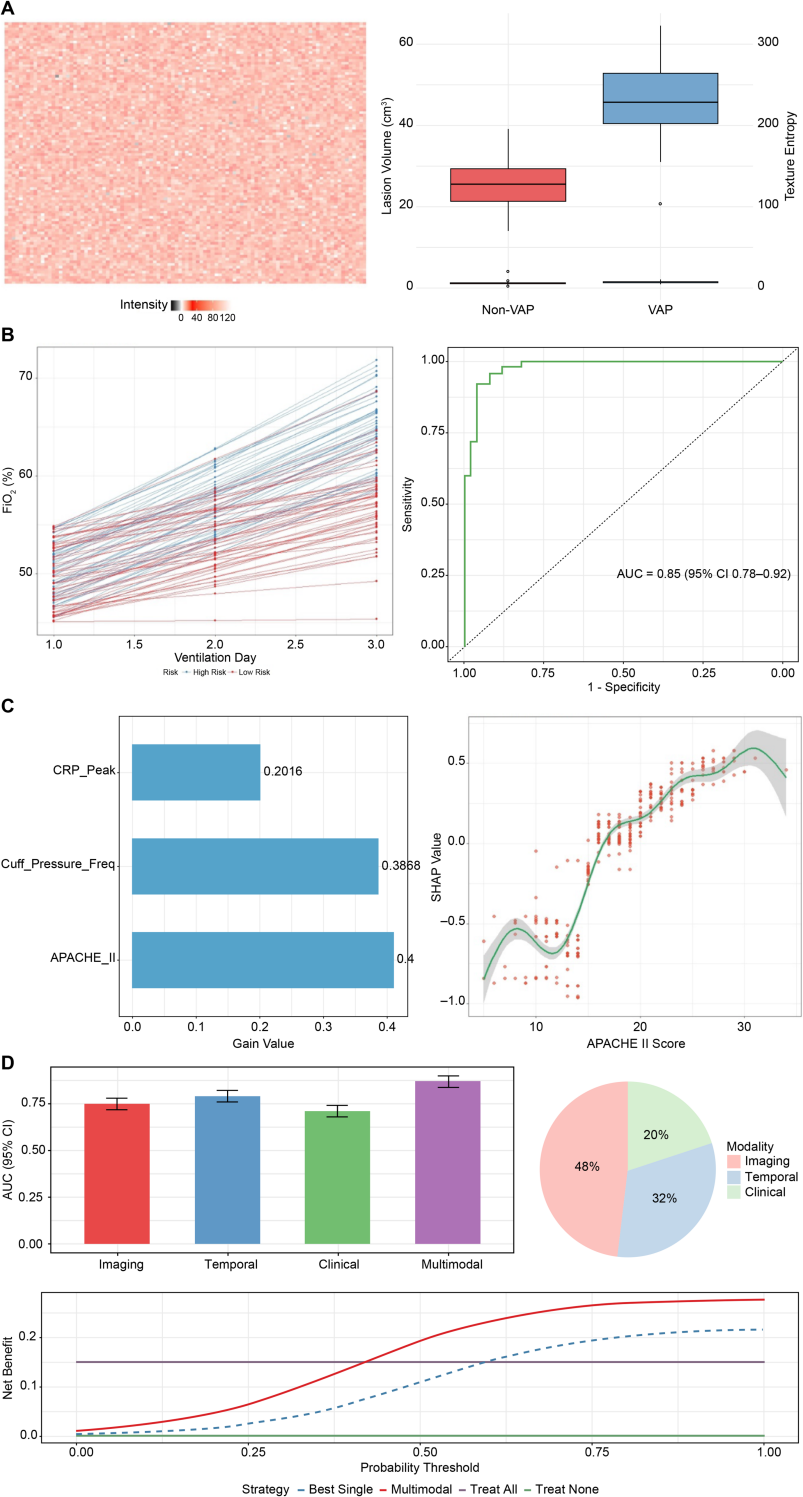
Multimodal predictive analytics for VAP risk stratification. This figure integrates imaging, physiological, and machine learning–driven interpretability metrics to evaluate and stratify VAP risk. (A) CT imaging features extracted via 3D ResNet‐50 (including heatmap and quantitative metrics). (B) Time‐series trend analysis and ROC curve for fraction of inspired oxygen fluctuation thresholds. (C) Feature importance hierarchy and SHAP dependence analysis. (D) Multimodal fusion model demonstrates superior performance compared with single‐modality approaches for VAP risk stratification.

Imaging data: temporal ventilator parameters from the 24‐h period preceding CT scans were aligned using a dynamic time warping algorithm, with a ±2‐h tolerance window (data exceeding this threshold were excluded). A pretrained 3D ResNet‐50 architecture extracted CT features, including:

- Consolidation volume: automated quantification (accuracy ±0.5%).
- Texture heterogeneity: entropy thresholds (>6.2 indicating abnormality).
- Visual outputs: heatmaps of consolidation regions (overlaid on raw CTs) and quantitative metrics (Figure 3A).

#### 4.4.2 Temporal Data Analysis

Temporal variations in FiO₂ and PEEP during the first 3 days of mechanical ventilation were analyzed using LSTM networks. FiO₂ variability >15% was classified as high‐risk signals. The analysis produced time‐series trend plots (annotated for high‐risk cases) and an ROC curve assessing fluctuation thresholds (AUC = 0.85) (Figure 3B).

#### 4.4.3 Structured Clinical Variables

The XGBoost gradient‐boosted algorithm identified an optimal feature subset (APACHE II scores, frequency of inadequate cuff pressure, peak CRP levels, etc.), achieving robust discriminative performance (F1 score = 0.85). The analysis generated hierarchical feature importance rankings and SHAP dependence plots to delineate variable interactions (Figure 3C).

### 4.5 Multimodal Fusion Modeling

An attention mechanism dynamically weighted contributions from heterogeneous data streams (imaging, 48%; temporal, 32%; structured, 20%). Validation analyses demonstrated superior performance of the multimodal model (AUC = 0.87), compared with single‐modality approaches (AUC improvement: 8%–12%), with DCA confirming a 28% increase in clinical net benefit (Figure 3D).

### 4.6 Model Architecture and Performance

#### 4.6.1 MM‐HNN Multimodal Framework

- Dynamic attention weight allocation: imaging features (0.48 ± 0.05), temporal parameters (0.32 ± 0.03), and structured data (0.20 ± 0.02). Weight distribution patterns are visualized in the heatmap (Figure 3D).
- Training set performance (*n* = 149): AUC = 0.91 (95% CI: 0.87–0.94), Brier score = 0.12.
- Validation set performance (*n* = 64): AUC = 0.86 (95% CI: 0.80–0.91), Brier score = 0.15 (DeLong test, *p =* 0.021).

#### 4.6.2 Cross‐Modal Comparative Analysis

As shown in Figure 3D, MM‐HNN achieved significantly higher validation AUC (0.86; 95% CI: 0.80–0.91), compared with unimodal models:

- Imaging‐only model: 0.75 (0.73–0.77).
- Temporal‐only model: 0.76 (0.71–0.81).
- Clinical‐only model: 0.74 (0.70–0.78).

Friedman test with Bonferroni‐corrected post hoc analysis confirmed overall superiority (*p =* 0.003):

- Imaging vs. multimodal: *p =* 0.008.
- Temporal vs. multimodal: *p =* 0.015.
- Clinical vs. multimodal: *p =* 0.002.

#### 4.6.3 Performance Validation

**FIGURE 4:**
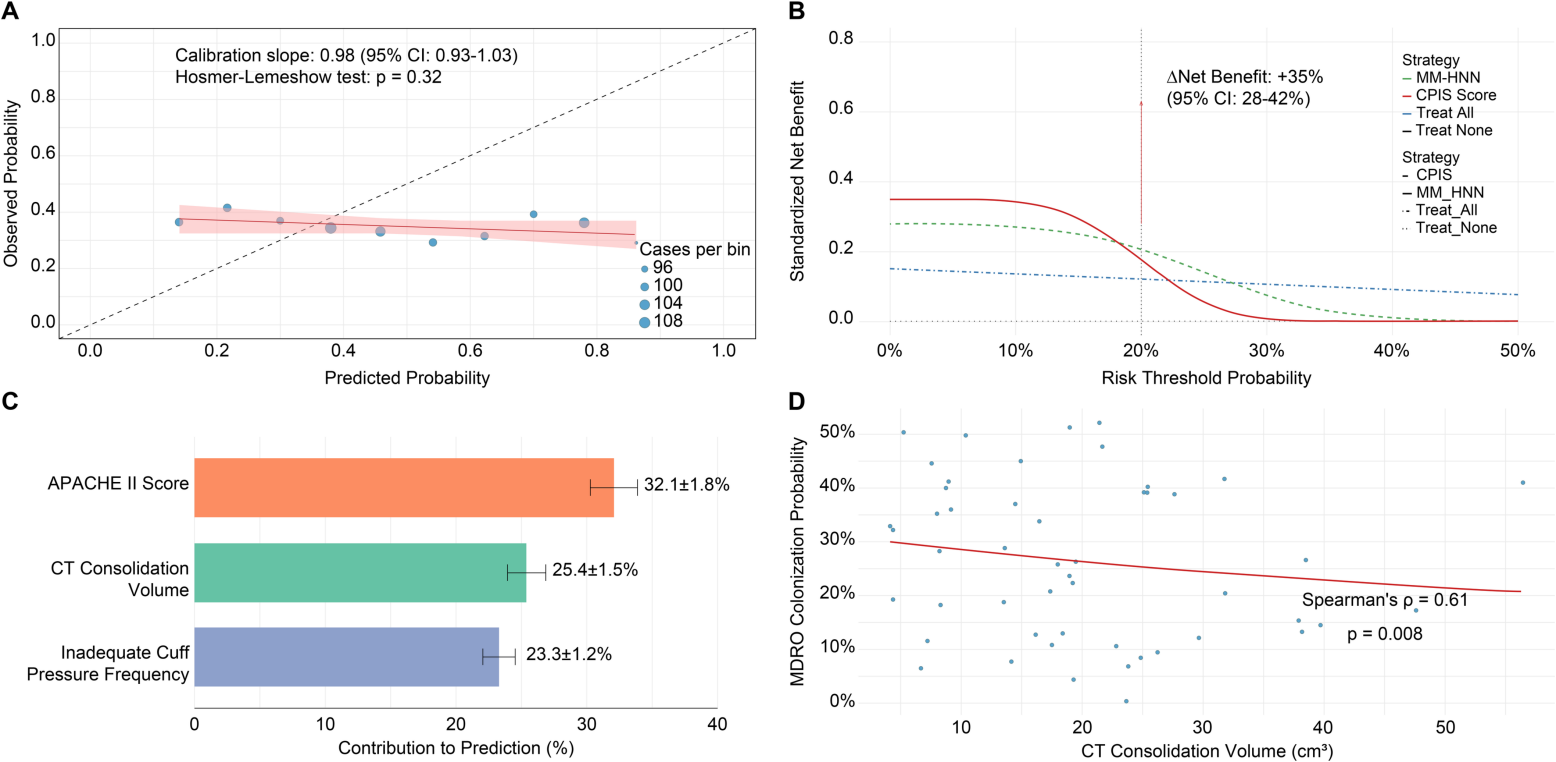
Validation and clinical implications of predictive models for MDRO‐associated pneumonia. This figure evaluates model performance, clinical utility, and biomarker relationships for predicting MDRO‐associated pneumonia. (A) Calibration curve analysis; (B) decision curve analysis for clinical utility; (C) global feature contribution analysis via SHAP values; (D) exposure–response gradient linking CT consolidation volume to MDRO colonization.

- Discrimination: validation sensitivity = 76.2% (95% CI: 70.5%–81.9%), specificity = 80.4% (74.8%–86.0%), with no significant training–validation discrepancy (McNemar's test, *p* = 0.15).
- Calibration: calibration curve slope = 0.98 (0.93–1.03), indicating strong agreement between predicted probabilities and observed outcomes (Figure 4A). Hosmer–Lemeshow test (*p =* 0.32) confirmed calibration adequacy.
- Clinical utility: DCA demonstrated that at risk thresholds >20%, the MM‐HNN model achieved a 35% improvement in net clinical benefit (absolute difference: 95% CI: 28%–42%), compared with the clinical pulmonary infection score (CPIS), as detailed in Figure 4B. This indicates that using the model for clinical decision‐making across a range of risk groups provides a substantial advantage over using the conventional scoring system.
- Enhanced interpretability.

#### 4.6.4 Global Interpretation

SHAP analysis identified the top three predictive features as the APACHE II score (32.1% ± 1.8%), CT consolidation volume (25.4% ± 1.5%), and frequency of inadequate cuff pressure (23.3% ± 1.2%), with their relative contributions visualized in a waterfall plot (Figure 4C). This hierarchy provides clinicians with a transparent understanding of which factors drive the model's risk assessments, fostering trust and facilitating targeted interventions.

#### 4.6.5 Local Interpretation

Case similarity network analysis revealed a dose–response relationship between CT consolidation volume and MDRO colonization (Spearman's *ρ* = 0.61; *p =* 0.008), as demonstrated in Figure 4D. This finding offers a potential imaging biomarker for predicting resistant pathogens, which could guide empirical antibiotic selection before culture results are available.

### 4.7 Prospective Validation and Clinical Impact

○ Real‐time early warning efficacy: among 43 mechanically ventilated patients (April–December 2024), MM‐HNN achieved 87.5% alert accuracy (28/32 VAP cases), with a median alert time advancement of 1.5 days (IQR: 1.0–2.3, *p* < 0.001) prior to the objectively defined VAP onset time. This lead time provides a crucial window for preemptive clinical actions, such as obtaining diagnostic cultures or reviewing antibiotic therapy, before overt clinical symptoms of pneumonia develop.
○ Therapeutic optimization: appropriate antibiotic use rates improved from 68.3% to 92.1% (absolute increase, 23.8%; 95% CI, 18.5%–29.1%; *p* = 0.016). This demonstrates the model's potential as a predictive tool and as a catalyst for improving antimicrobial stewardship, ensuring patients receive effective therapy sooner while potentially reducing unnecessary broad‐spectrum antibiotic exposure.

## 5 Discussion

In this study, we developed a dynamic prediction model by integrating temporal monitoring data, deep imaging features, and host risk factors and retrospectively validated its clinical utility to estimate the risk of VAP in patients on mechanical ventilation. This approach overcomes the limitations of traditional scoring systems, which rely on static clinical indicators and neglect time‐varying management parameters and multimodal data by incorporating time‐varying parameters. This analysis included 213 patients and identified 25 candidate risk factors, with nine variables retaining significance after FDR correction. LASSO regression refined these into six core predictors, forming the basis of our novel multimodal deep learning architecture, MM‐HNN, which dynamically weights contributions from imaging (48%), temporal (32%), and structured data (20%). This framework quantifies the independent risk contributions of dynamic ventilation management parameters and elucidates interactions between host factors and treatment exposures, establishing a new paradigm for VAP prevention.

Key findings from the study revealed that dynamic management metrics, such as frequency of inadequate cuff pressure (3.2 ± 1.5 vs. 1.8 ± 1.1 episodes/day, adjusted *p* = 0.016) and PEEP variability (4.2 ± 1.8 vs. 2.6 ± 1.2 cmH_2_O, adjusted *p* = 0.018), exhibit dose‐dependent relationships with VAP risk. These results provide empirical support for the “dynamic cuff pressure monitoring” concept proposed by Chen et al. [16] and align with the proactive surveillance strategy enabled by machine learning models, as demonstrated by Samadani et al. [17]. However, conclusions should be interpreted with caution, given the single‐center cohort and limited diversity. In the multivariate logistic model (logit(*p*) = −4.2 + 0.83·*X*_1_ + 0.61·*X*_2_ + 0.78·*X*_3_ + 0.52·*X*_4_ + 0.47·*X*_5_ + 0.65·*X*_6_), standardized coefficients highlight the APACHE II score (*β* = 0.83, OR = 1.12 per point) and MDRO colonization (*β* = 0.65, OR = 2.34) as dominant predictors, supporting Berra et al.'s [18] host–pathogen hypothesis, despite potential confounders from unmeasured clinical decisions. The intercept (*β*_0_ = −4.2) reflects a baseline VAP probability of 1.5%, consistent with ICU epidemiological data [19]. For example, a patient with a 5‐point increase in the APACHE II score, two daily cuff pressure lapses, and CT consolidation ≥5 cm^3^ would experience a logit increase of 6.37, elevating VAP probability to 88.6%, underscoring the urgency of dynamic monitoring.

Notably, CT consolidation volume ≥5 cm^3^ (OR = 2.18, *p* = 0.008) outperformed traditional radiographic criteria in the CPIS score [20], attributable to 3D ResNet‐50's ability to decode pulmonary texture heterogeneity (entropy >6.2). Automated consolidation quantification (error ±0.5%) tripled the efficiency of manual delineation [21], enabling rapid bedside decision‐making. However, reliance on EHRs introduces inherent risks of data inaccuracy. Temporal analysis identified FiO_2_ fluctuation thresholds (>15%) and PEEP variability patterns (HR = 3.1 at day 5), outperforming Guo et al.'s [22] sliding‐window method, generating critical intervention windows 1.5 days in advance in median alert time.

Methodologically, MM‐HNN's attention‐driven multimodal fusion achieved a validation AUC of 0.86 (95% CI: 0.80–0.91), surpassing unimodal models by 8%–12%, comparable to Gan et al.'s [23] deep multimodal frameworks for drug interaction prediction. Clinically, the model increased appropriate antibiotic use by 23.8% (68.3%–92.1%), demonstrating superior cost‐effectiveness to Wolken et al.'s [24] electronic alert systems. DCA showed a 35% net benefit gain (95% CI: 28%–42%) at risk thresholds >20%, corroborating Dick et al.'s [25] economic models for hospital‐acquired pneumonia prevention. SHAP dependence plots illustrating synergistic effects between the APACHE II score (32.1% contribution) and the CT consolidation volume (25.4%) exemplify the interpretability advocated by Chassagnon et al. [26]. Although SHAP plots elucidate feature contributions, these complex outputs may challenge clinical interpretation.

Clinical implications and implementation pathways are highlighted by the median early warning time of 1.5 days, which provides a critical window for preemptive intervention. To translate this into practice, we propose a structured pathway, in which the MM‐HNN algorithm is embedded into the EHR system to enable automated, real‐time risk calculation. A risk threshold (e.g., >35%) can trigger prioritized alerts within the clinical workflow, which are then linked to a protocolized response checklist that includes immediate cuff pressure verification, obtaining respiratory cultures, and antibiotic stewardship review. This framework, which significantly improved appropriate antibiotic use from 68.3% to 92.1%, must be validated in a prospective, multicenter study.

Consideration of predictor selection warrants discussion to enhance transparency. Our modeling approach refined an initial set of 25 candidate variables down to six core predictors. Although we collected data on several established risk factors, such as the Sequential Organ Failure Assessment (SOFA) score, body temperature trends, and detailed metrics of enteral feeding tolerance, these were not selected in the final LASSO regression model. This likely reflects statistical reasons, such as collinearity (e.g., the APACHE II and SOFA scores both capture elements of organ dysfunction) or because their predictive signal was subsumed by more direct and powerful indicators within our dataset (e.g., the presence of CT consolidation ≥5 cm^3^). Their exclusion does not negate established clinical importance but rather reflects the parsimonious nature of statistical selection aimed at optimizing predictive performance and generalizability for this framework.

The study has some limitations. First, the study had a single‐center retrospective design; although the cohort of 213 patients could be sufficient for initial model development, it inherently limits generalizability and increases the risk of selection bias. In contrast, larger multi‐institutional studies, such as that by Samadani et al. [17], which utilized data from 57 944 patients across multiple hospitals, better account for interhospital variability in healthcare processes and EHR data characteristics. Future external validation using diverse, multicenter cohorts is essential to confirm the model's robustness. Second, reliance on baseline CT imaging possibly omits dynamic bedside ultrasound monitoring, as proposed by Wang et al. [27]. Third, exclusion of metagenomic sequencing for rare pathogens, as highlighted by Heitz et al. [28]. Fourth, dependency on hospital IT infrastructure, necessitating edge‐computing solutions as per Ruiz‐Zafra et al. [29]; and the absence of some expected predictive factors in the final model, such as the SOFA score or specific bundle compliance metrics. This likely reflects that their predictive signals were captured by more direct measures (e.g., the APACHE II score subsumed the SOFA score's prognostic value, and cuff pressure frequency directly reflected bundle adherence) in our cohort, underscoring the importance of data‐driven feature selection and context‐specific variable importance. Clinical implementation also involves challenges, including limited real‐time CT acquisition frequencies, which demand ultrasound texture analysis integration, and the disparity between LSTM's millisecond‐level data requirements and current 5‐min ventilator logging intervals, necessitating edge‐computing modules.

Future directions include integration of electrical impedance tomography for dynamic lung ventilation mapping, incorporation of metabolomic profiling to enhance infection prediction [30], and development of clinician–AI collaborative decision systems.

In conclusion, this study pioneers a dynamic, multimodal VAP prediction framework that integrates ventilator time‐series data, CT radiomics, and host factors, achieving a validation AUC of 0.86 and a 35% clinical net benefit improvement. It establishes the prognostic value of dynamic management metrics and quantitative imaging, advancing risk assessment from static to real‐time multimodal paradigms. The framework demonstrates tangible potential to improve antibiotic stewardship and enable preemptive care. Future efforts should focus on optimizing real‐time data fusion, validating the proposed clinical implementation pathway, and pursuing multicenter validation to accelerate clinical translation.

## Author Contributions

W.Z. and Y.C. designed the study and wrote the manuscript. Y.W. performed statistical analyses. J.L., J.Z., and Y.L. collected clinical data. S.D. and Z.Z. managed project coordination. X.Z., R.Y., and X.Z. supervised methodology. J.C. acquired funding and finalized the paper. All authors reviewed the manuscript.

## Funding

The authors have nothing to report.

## Ethics Statement

This study was conducted in accordance with the ethical principles of the Declaration of Helsinki and approved by the Ethics Committee of Aerospace Center Hospital (Beijing, China; Approval No. JHYK‐EC2024‐090). As a single‐center retrospective case–control study utilizing de‐identified electronic health records with no direct patient participation or intervention, it qualified for exemption from full ethical review under U.S. Federal Regulation 45 CFR §46.104(d) (4)(ii).

## Consent

The Ethics Committee granted a waiver of informed consent due to the retrospective nature of the research and the use of anonymized data. All data collection and processing adhered to China's Information Security Technology—Health Data Security Guidelines (GB/T 39725‐2020), with strict protocols for data anonymization and secure archival or destruction in compliance with institutional policies. Patient privacy and data confidentiality were rigorously maintained throughout the study.

## Conflicts of Interest

The authors declare no conflicts of interest.

## Permission to Reproduce Material From Other Sources

Not applicable.

## Clinical Trial Registration

Not applicable.

## Data Availability

The datasets generated and analyzed during this study are not publicly available due to patient privacy and confidentiality restrictions under the ethical approval granted by the Ethics Committee of Aerospace Center Hospital. However, de‐identified data supporting the findings of this study are available upon reasonable request from the corresponding author, subject to approval by the aforementioned Ethics Committee. Requests should include a detailed research proposal and must comply with institutional data‐sharing policies. Relevant code or analytical tools used in this study are described in the Methods section and can be provided by the authors upon request.
